# A Case of Rapid Development of Methicillin‑Resistant Staphylococcus aureus Mechanical Aortic Root Abscess Despite Appropriate Antibiotic Use

**DOI:** 10.7759/cureus.17494

**Published:** 2021-08-27

**Authors:** Sanjay Chandrasekhar, Dae Hyun Lee, Nidhi Patel, Emmanuel Bassily, Allan Chen

**Affiliations:** 1 Cardiology, University of South Florida, Tampa, USA; 2 Cardiology, Orlando Health Heart Institute, Orlando, USA

**Keywords:** infective endocarditis, aortic root abscess, transesophageal echocardiogram, staphylococcus aureus endocarditis, prosthetic valve infective endocarditis

## Abstract

A 74-year-old male with a past medical history of mechanical aortic valve replacement presented with abdominal pain and fever. Blood cultures revealed methicillin-resistant *Staphylococcus aureus* (MRSA) and the patient was started on target antibiotics. Initial transthoracic echocardiogram and transesophageal echocardiogram (TEE) did not show any vegetations or significant valvular regurgitation. No other sources of infection were identified. Five days after the initial TEE, a repeat TEE revealed new areas of thickening and echolucency seen anterior to the mechanical aortic valve, suggestive of aortic root abscess (AoRA). It also extended down the mitral-aortic intervalvular fibrosa and was associated with mitral valvular vegetation.

Due to worsening clinical status and persistent bacteremia on appropriate antibiotics, a high index of suspicion for infective endocarditis (IE) remained after the initial TEE. As such, the repeat TEE was obtained only five days after and demonstrated clear evidence of rapidly growing endocarditis and abscess formation. This case uniquely demonstrates how rapid MRSA endocarditis may progress and emphasizes its high mortality. This case highlights the importance of a low threshold for repeat imaging when the index of suspicion for endocarditis remains high despite negative imaging.

## Introduction

Infective endocarditis (IE) is an uncommon, yet potentially lethal disease with increasingly complex epidemiology. The overall incidence of IE secondary to *Staphylococcus aureus* has grown and is now the most common causative organism in most of the industrialized world [1]. In particular, methicillin-resistant *Staphylococcus aureus* (MRSA) infective endocarditis has high morbidity and mortality. IE is typically visualized on transthoracic echocardiography (TTE) and transesophageal echocardiogram (TEE). TEE generally allows for a more detailed visualization of valves and potential pathology. Aortic root abscesses are well described in MRSA IE; however, the length of time for which aortic root abscesses can develop remains unclear. Most cases have not been shown to progress in a matter of days; however, if initial testing is negative, a high index of suspicion should remain for repeat imaging in the appropriate clinical context, given the high mortality that can underlie IE and valvular abscesses and significant differences in treatment recommendations. Repeating TEE in these cases of high clinical suspicion remains a Class 1 recommendation in both American and European guidelines. In this report, we describe a case of rapid onset of an MRSA aortic root abscess despite negative initial TEE.

## Case presentation

**Figure 1:**
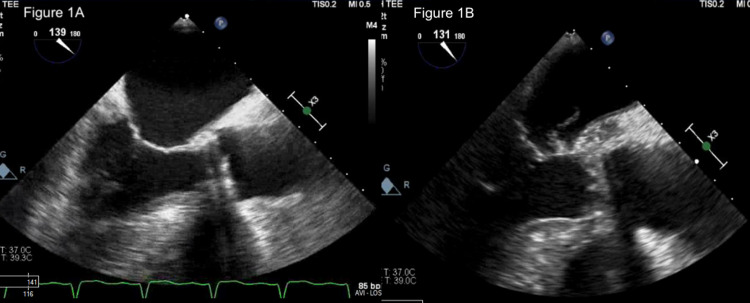
Transesophageal echocardiogram at the mid-esophageal view at baseline (A) and at five days (B).

A 74-year-old male presented with abdominal pain and fever for several days who had a past medical history of mechanical aortic valve replacement seven years ago, diabetes mellitus, and morbid obesity. Initial computed tomography (CT) scan of chest, abdomen, and pelvis showed possible cellulitis, inferior vena cava (IVC) filter with strut penetration, and lung consolidation. Blood cultures were drawn and broad-spectrum antibiotics with vancomycin, aztreonam, and metronidazole were initiated. Initial blood cultures grew MRSA. A transthoracic echocardiogram was performed to screen for IE. Initial transthoracic echocardiogram (TTE) and subsequent transesophageal echocardiogram (TEE) were negative for signs of vegetation or valvular abnormalities. The patient continued to progress with worsening respiratory compromise and oliguric acute kidney injury that required treatment in the intensive care unit. Repeat blood cultures despite appropriate antibiotics demonstrated persistent MRSA bacteremia. Four days after the initial TEE, the decision was made to repeat TEE given high clinical suspicion of IE. Repeat TEE illustrated new areas of thickening and echolucency posterior to the mechanical aortic valve extending down the mitral-aortic intervalvular fibrosa that was suggestive of aortic root abscess (Figure 1).

The patient was deemed an extremely high-risk surgical candidate with an overall worsening condition in the ICU and ultimately made comfort measures only. The patient subsequently passed away.

## Discussion

Infective endocarditis is commonly seen in MRSA bacteremia. Early clinical suspicion is vital, as is the early initiation of appropriate antibiotics. If there is a lack of source control, as in our patient, then it is critical to repeat imaging to look for endocarditis or abscess as this can impact treatment. Classically, diagnosis can be made with modified Duke’s criteria, which remains a Class I American Heart Association (AHA) recommendation [1,2]. This patient had one major and two minor modified Duke’s criteria, which raised suspicion for IE.

Echocardiography remains the first-line imaging modality of choice in all suspected cases in both the European Society of Cardiology (ESC) and the AHA 2015 guidelines [1,3]. Echocardiography in cases of isolated *Staphylococcus aureus* bacteremia without a clear source can be considered even without additional Duke criteria as a Class IIa recommendation [2,3]. Initial TTE surveillance in this patient was negative with progression to TEE given high suspicion, which is a Class I recommendation [1,3]. Despite negative initial imaging, worsening clinical status and persistent bacteremia should elicit repeat imaging [4]. Repeat imaging within three to seven days in this context is a Class I indication by both AHA and ESC guidelines [1-3]. Vegetations, abscess cavities, or fistulous tracts may develop or become detectable at the time of repeat imaging [1]. If there is a continued suspicion for paravalvular infection with inadequate TTE/TEE images, a cardiac CT can be considered with a Class IIa AHA recommendation [2]. Additional Class I indications for repeat TEE would be in patients with initial positive TEE and development of worrisome clinical features including worsening heart failure symptoms, change in a cardiac murmur, and new atrioventricular blocks or other arrhythmias despite appropriate antibiotic therapy [1].

Repeat TEE illustrated aortic root abscess in the context of the history of mechanical aortic valve replacement. Valvular abscess remains a Class I indication for early surgery in order to achieve source control [2]; unfortunately, the worsening clinical condition placed the patient at a prohibitively high surgical risk.

This case illustrates how aggressive *Staphylococcus aureus* bacteremia can be seen even with the early initiation of appropriate antibiotics. The patient initially had no evidence of endocarditis or paravalvular abscess on TTE/TEE and within four days on antibiotics developed a significant aortic root abscess. Paravalvular abscesses associated with *Staphylococcus aureus *bacteremia have high mortality; a retrospective review illustrated a 31% in-hospital mortality and 38% one-year mortality with these patients [5]. The initial medical treatment with antibiotics may not be responsive to vancomycin and may need other lines of therapies such as daptomycin or linezolid [6]. Only a few cases of rapid abscess formation to this degree have been reported; only one on our review of the literature with aortic root abscess [7]. Rapid abscess formation around the mitral valve has additionally been reported [8].

## Conclusions

Despite appropriate antibiotics, *Staphylococcus aureus* bacteremia can remain aggressive and a high index of suspicion for surveillance for IE is critical. Considering the lack of improvement and persistence of cultures, early repeat imaging with TEE and possibly even cardiac CT is important. As this case illustrates, the rapid development of lethal endocarditis and abscess can occur over the time span of days despite appropriate antibiotics. Serial imaging studies and early surgery may be necessary given aggressive infections and known high mortality rates.
